# Imputation provides an opportunity to study filaggrin ( *FLG*) null mutations in large population cohorts that lack bespoke genotyping

**DOI:** 10.12688/wellcomeopenres.17657.1

**Published:** 2022-02-01

**Authors:** Lavinia Paternoster, Ashley Budu-Aggrey, Sara J. Brown

**Affiliations:** 1 1. MRC Integrative Epidemiology Unit, Bristol Medical School, Population Health Sciences, The University of Bristol, Bristol, BS8 2BN, UK; 2 2. Centre for Genomics and Experimental Medicine, Institute for Genetics and Cancer, University of Edinburgh, Edinburgh, EH4 2XU, UK

**Keywords:** Filaggrin, genotyping, ALSPAC, UK Biobank

## Abstract

**Background:** Low frequency mutations within the filaggrin ( *FLG*) gene are established genetic risk factors for atopic dermatitis. Studies of *FLG* have typically used sequencing or bespoke genotyping. Large-scale population cohorts with genome-wide imputed data offer powerful genetic analysis opportunities, but bespoke *FLG* genotyping is often not feasible in such studies. Therefore, we aimed to determine the quality of selected *FLG* null genotype data extracted from genome-wide imputed sources, focussing on UK population data.

**Methods:** We compared the allele frequencies of three *FLG* null mutations (R501X, R2447X and S3247X) in directly genotyped and genome-wide imputed data in the ALSPAC cohort. Logistic regression analysis was used to test the association of atopic dermatitis with imputed and genotyped *FLG* null mutations in ALSPAC and UK Biobank to investigate the usefulness of imputed *FLG* data.

**Results:** The three *FLG* null mutations appear to be well imputed in datasets that use the Haplotype Reference Consortium (HRC) for imputation (0.3% discordance compared with directly genotyped data). However, a greater proportion of null alleles failed imputation compared to wild-type alleles. Despite the calling of *FLG* mutations in imputed data being imperfect, they are still strongly associated with atopic dermatitis (p-values between 7x10 ^-10^ and 5x10 ^-75^ in UK Biobank).

**Conclusions:** HRC imputed data appears to be adequate for UK population-based genetic analysis of selected *FLG* null mutations.

## Abbreviations

AD                Atopic dermatitis

ALSPAC      Avon Longitudinal Study of Parents and Children

CI                 Confidence interval

*FLG*             Gene encoding filaggrin

HRC            Haplotype Reference Consortium

KASP          Competitive (Kompetative) allele-specific PCR

OR              Odds ratio

## Introduction

The gene encoding filaggrin ( *FLG*) has long been established as an important genetic risk factor for atopic dermatitis (AD) ^1, 2^. Several low frequency variants that truncate the protein product (loss-of-function, null mutations) have been identified and the most common are regularly genotyped in studies of AD. These mutations were identified in sequencing studies ^3^, and specific TaqMan® genotyping assays ^1^ have been designed and used, and more recently KASP ^TM^ assays have been validated for genotyping these mutations in population epidemiological studies ^4^. With the rapid expansion of genome-wide genotyping and imputation procedures to generate consistent genome-wide data in large cohort studies, we wanted to investigate if such imputation procedures are sufficiently accurate to be used for generating genotype information for the most common *FLG* null mutations. If genome-wide imputation can recapitulate *FLG* null mutation information then this would facilitate the study of this gene in some very large population cohort studies without bespoke genotyping, including the UK Biobank (N=500,000 participants) and 23andMe (N=2 million).

Here we investigate the imputation quality of three *FLG* null mutations in 2 well characterised cohorts: The Avon Longitudinal Study of Parents and Children (ALSPAC, HRC imputation, N=~5000) and UK Biobank (HRC+UK10K imputation, N=~330,000), to determine whether use of imputed *FLG* genotypes is appropriate in epidemiological studies.

In the ALSPAC cohort we have undertaken bespoke genotyping of 4 *FLG* mutations using KASP ^TM^ (R501X, 2282del4, R2447X and S3247X). Also available for the same individuals are genome-wide imputed data using the Haplotype Reference Consortium (HRC.r1.1, 2016 ^5^). The deletion 2282del4 is not captured by the HRC imputation panel, therefore in this study we compared imputed data for the 3 other mutations with the bespoke genotype data to investigate whether the associations with AD using different genetic data sources are reproducible. We also investigated the association between AD and imputed *FLG* variants from the UK Biobank cohort.

## Methods

### ALSPAC cohort

Enrolment of the ALSPAC cohort has been fully described previously ^6, 7^. Briefly, pregnant women resident in Avon, UK with expected dates of delivery 1st April 1991 to 31st December 1992 were invited to take part in the study. The initial number of pregnancies enrolled was 14,541. Of these initial pregnancies, there were 14,676 foetuses, resulting in 14,062 live births and 13,988 children who were alive at 1 year of age. When the oldest children were approximately 7 years of age, an attempt was made to bolster the initial sample with eligible cases who had failed to join the study originally. As a result, the total sample size for analyses using any data collected after the age of seven is 15,454 pregnancies, resulting in 15,589 foetuses. Of these individuals, 14,901 were alive at 1 year of age. The study website contains details of all the data that is available through a fully searchable data dictionary and variable search tool. Ethical approval for the study was obtained from the ALSPAC Ethics and Law Committee and the Local Research Ethics Committees.

The children have been followed up with regular questionnaires and clinic visits. Data collected from questionnaires was used to classify children as AD cases or controls. When the children were approximately 81, 91, 103 months, 10, 13, 14 years, parents were asked the following questions [possible answers]:

1. Has your child in the past 12 months had eczema? [Yes and saw a Dr; Yes, but did not see a Dr; No]
2. Has a doctor ever actually said that your child has eczema? [yes; no] (asked at 10 & 14 years)

We defined AD cases as the children whose parents answered “Yes and saw a Dr” to Q1 or “yes” to Q2. We defined controls as the children who were not a case and whose parents answered “No” to Q2 at 14 years.

### ALSPAC - Genetic data

Four *FLG* mutations (R501X, 2282del4, R2447X, S3247X) were genotyped in the ALSPAC mothers and children by LGC Genomics (Middlesex, UK) using KASP ^TM^ genotyping technology. Genotypes were available for 10,197 children and 8,811 mothers.

Two combined null genotype variables were generated using these data. One included all 4 genotyped variants, and a second that excluded 2282del4 to allow comparison with the imputed data, where this variant was not available. For each of these *FLG* combined null variables, presence of any one *FLG* mutation was sufficient to class that individual as filaggrin haploinsuffcient. Individuals with no missing data and no *FLG* mutations were categorised as normal wild-type genotype.

The ALSPAC genome-wide data has been described previously ^8^. Briefly, ALSPAC children were genotyped using the Illumina HumanHap550 quad chip genotyping platforms by 23andme subcontracting the Wellcome Trust Sanger Institute, Cambridge, UK and the Laboratory Corporation of America, Burlington, NC, US. The resulting raw genome-wide data were subjected to standard quality control methods. Individuals were excluded on the basis of sex mismatches; minimal or excessive heterozygosity; disproportionate levels of individual missingness (> 3%) and insufficient sample replication (IBD < 0.8). Population stratification was assessed by multidimensional scaling analysis and compared with Hapmap II (release 22) European descent (CEU), Han Chinese, Japanese and Yoruba reference populations; all individuals with non-European ancestry were removed. SNPs with a minor allele frequency of < 1%, a call rate of < 95% or evidence for violations of Hardy-Weinberg equilibrium (p < 5x10 ^-7^) were removed. Cryptic relatedness was measured as proportion of identity by descent (IBD > 0.1). Related subjects that passed all other quality control thresholds were retained during subsequent phasing and imputation. 9,115 subjects and 500,527 SNPs passed these quality control filters.

ALSPAC mothers were genotyped using the Illumina human660W-quad array at Centre National de Génotypage (CNG) and genotypes were called with Illumina GenomeStudio. PLINK (v1.07) was used to carry out quality control measures on an initial set of 10,015 subjects and 557,124 directly genotyped SNPs. SNPs were removed if they displayed more than 5% missingness or a Hardy-Weinberg equilibrium p-value of less than 1.0x10 ^-6^. Additionally, SNPs with a minor allele frequency of less than 1% were removed. Samples were excluded if they displayed more than 5% missingness, had indeterminate X chromosome heterozygosity or extreme autosomal heterozygosity. Samples showing evidence of population stratification were identified by multidimensional scaling of genome-wide identity by state pairwise distances using the four HapMap populations as a reference, and then excluded. Cryptic relatedness was assessed using an identical by descent (IBD) estimate of more than 0.125 which is expected to correspond to approximately 12.5% alleles shared IBD or a relatedness at the first cousin level. Related subjects that passed all other quality control thresholds were retained during subsequent phasing and imputation. 9,048 subjects and 526,688 SNPs passed these quality control filters.

The 477,482 SNP genotypes in common between the sample of mothers and sample of children were combined. SNPs with genotype missingness above 1% due to poor quality were removed (11,396 SNPs) and a further 321 subjects were removed due to potential ID mismatches. This resulted in a dataset of 17,842 subjects containing 6,305 duos and 465,740 SNPs (112 were removed during liftover and 234 were out of HWE after combination). Haplotypes were estimated using ShapeIT (v2.r644) which utilises relatedness during phasing. The phased haplotypes were then imputed to the Haplotype Reference Consortium (HRCr1.1, 2016) panel of approximately 31,000 phased whole genomes. The HRC panel was phased using ShapeIt v2, and the imputation was performed using the Michigan imputation server. R ^2^ imputation quality measures were available for all imputed variants.

This gave 8,237 eligible children and 8,196 eligible mothers with available genotype data after exclusion of related subjects using cryptic relatedness measures described previously.

Data on the 3 imputed *FLG* variants (R501X:rs61816761, R2447X:rs138726443 & S3247X:rs150597413) were extracted from this data.

Best-guess calls and genotype probabilities for the three possible genotypes at each variant were available for all individuals. Best guess genotypes were generated using a hard-call-threshold of 0.1 in Plink. These 3 best-guess genotypes were also combined into an overall imputed *FLG* combined null genotype, where presence of any one *FLG* mutation was sufficient to class that individual as filaggrin haploinsufficient. Individuals with no missing data and no *FLG* mutations were categorised as wild-type.

### UK Biobank cohort

UK Biobank is a population-based health research resource consisting of approximately 500,000 people, aged between 38 years and 73 years, who were recruited between the years 2006 and 2010 from across the UK ^9^. Particularly focused on identifying determinants of human diseases in middle-aged and older individuals, participants provided a range of information (such as demographics, health status, lifestyle measures, cognitive testing, personality self-report, and physical and mental health measures) via questionnaires and interviews; anthropometric measures, BP readings and samples of blood, urine and saliva were also taken (data available at www.ukbiobank.ac.uk). A full description of the study design, participants and quality control (QC) methods have been described in detail previously ^10^. UK Biobank received ethical approval from the North West Research Ethics Committee (REC reference for UK Biobank is 11/NW/0382).

Individuals were defined as having atopic dermatitis (AD) based on their response during a verbal interview with a trained member of staff at the assessment centre. Participants were asked to tell the interviewer which serious illnesses or disabilities they had been diagnosed with by a doctor and were defined as AD cases if this disease was mentioned. Disease information was also obtained from the Hospital Episode Statistics (HES) data extract service where health-related outcomes had been defined by International Classification of Diseases (ICD)- 10 code L20. Additionally, anyone who had had answered “yes” to “Has a doctor ever told you that you have hay fever, allergic rhinitis or eczema”, were excluded from the AD controls.

### UK Biobank – Genetic data

Overall, 49,979 individuals were genotyped using the UK BiLEVE array and 438,398 using the UK Biobank axiom array (n=488,377 total). Pre-imputation QC, phasing and imputation are described elsewhere ^11^. In brief, prior to phasing, multiallelic SNPs or those with MAF ≤1% were removed. Phasing of genotype data was performed using a modified version of the SHAPEIT2 algorithm ^12^. Genotype imputation to a reference set combining the UK10K haplotype and HRC reference panels ^13^ was performed using IMPUTE2 algorithms ^14^. MAF and Info scores were recalculated on the derived ‘European’ subset. Additional quality control exclusions were applied to the data as described previously ^15^. Briefly, individuals with sex-mismatch, sex chromosome aneuploidy, outlying degrees of heterozygosity and/or missingness and related individuals were excluded. For this analysis we also restricted the sample to individuals of white British ancestry who self-report as “White British” and who have very similar ancestral backgrounds according to the PCA, as described by Bycroft ^11^. This resulted in 337,076 individuals with available genetic imputed data.

Data on the 3 imputed *FLG* variants (R501X:rs61816761, R2447X:rs138726443 & S3247X:rs150597413) were extracted from this data.

Best-guess calls and genotype dosages were available for all individuals. Best guess genotypes were generated using a hard call threshold of 0.1. These 3 best-guess genotypes were also combined into an overall imputed *FLG* combined null genotype, where presence of any one *FLG* mutation was sufficient to class that individual as filaggrin haploinsufficient. Individuals with no missing data and no *FLG* mutations were categorised as wild-type.

### Concordance of KASP ^TM^ and imputed genetic data

Minor allele frequencies for KASP ^TM^ genotyped and best-guess imputed data were calculated in R (version 3.6.1) from the genotype call frequencies. Minor allele frequencies for uncertain imputation data (i.e. genotype probabilities or dosages) were extracted directly from the relevant imputation output for each cohort.

Concordance of genotypes at an individual level between the KASP ^TM^ genotyped and imputed data was assessed for ALSPAC by producing contingency tables in R. Proportions were then calculated to assess the overall discordance and the proportions mis-called or missing for particular categories.

### Associations between genotypes and AD in ALSPAC

In ALSPAC, associations between AD and individual KASP ^TM^ genotypes was conducted using general linear modelling in R (adjusting for sex) and assuming an additive model. Associations between AD and imputed variants was conducted using SNPTEST (adjusting for sex and 10 principal components) and assuming an additive model, using the genotype probabilities (and the em algorithm) to account for the uncertainty in the genotype calling.

Associations between AD and *FLG* combined null genotype for both KASP genotyped and imputed data was conducted using general linear modelling in R (adjusting for sex). The KASP genotyped combined null genotype analyses were conducted including and excluding the 2282del4 variant, for comparison.

In UK Biobank, associations between AD and imputed variants were conducted in PLINK 2.0 using general linear modelling, assuming an additive model and adjusting for sex, chip and 10 principal components. This was performed with genotype dosages to account for the uncertainty in the genotype calling. Associations between AD and *FLG* combined null genotype was also conducted using general linear modelling in R (adjusting for sex).

## Results and discussion

Table 1 shows the allele frequencies of *FLG* R501X, R2447X and S3247X from the ALSPAC KASP ^TM^ data versus the HRC imputed data. The UK Biobank HRC frequencies are also shown for comparison. The allele frequencies of the complete imputed data are consistent between the two ALSPAC genetic datasets; the UK Biobank frequencies are also in keeping with expected values. However, of note, the frequencies calculated from only those individuals for whom a confident genotype call could be made are lower for all 3 SNPs in the ALSPAC data and for R501X in the UK Biobank data, suggesting that those with mutations are disproportionately harder to call from the imputed data than those with homozygous wild-type genotype. The proportion of individuals who carry at least 1 *FLG* null mutation at any of these positions (combined null genotype) as inferred from imputed data is slightly lower (4%) than when the genotyped data is used (6%), and it is important to note that omission of 2282del4 from the imputed data means that the total percentage of individuals with *FLG* haploinsufficiency (combined null genotype) is substantially lower than the percentage defined by genotype data including all 4 null mutations (10%) ( Table 1).

**Table 1.** Allele frequencies and imputation quality of *FLG* null mutations in ALSPAC (as measured by KASP ^TM^ genotyping and HRC imputation) and in UK Biobank (HRC-UK10K imputation only).

*FLG* variants	rs ID	ALSPAC KASP	ALSPAC HRC imputation		UK Biobank imputation	
		Freq	Freq (confident calls)	R ^2^	Freq (confident calls)	info
R501X	rs61816761 (T)	0.021	0.023 (0.015)	0.82	0.023 (0.016)	0.89
R2447X	rs138726443 (T)	0.004	0.005 (0.002)	0.73	0.005 (0.005)	1
S3247X	rs150597413 (A)	0.003	0.003 (0.001)	0.79	0.004 (0.004)	1
Combined null genotype	3 mutations *3 plus 2282del4	0.06 (0.10) *	- (0.04)	-	- (0.05)	-

Frequencies displayed are for the rare allele at each position (the allele is shown in the rsID column). “Freq” is the minor allele frequency calculated from all individuals – but accounts for the uncertainty of individual genotype calls. The minor allele frequency calculated only from the individuals with “confident calls” (uncertainty<=0.1) are also shown. For the combined null genotype status the freq. columns show the proportion of individuals that carry at least 1 *FLG* null mutation. *For the KASP genotyped data, two proportions are given, one counting only the 3 SNP variants and the second in brackets also includes the 2282del4 mutation. “R ^2^” and “info” denote the imputation quality measures estimated during the imputation procedures: R ^2^ is the imputation quality score reported by Minimac and info is reported by IMPUTE software. *FLG*, filaggrin; HRC, Haplotype Reference Consortium; KASP, Competitive (Kompetative) allele-specific PCR.

The imputation quality scores (reported in Table 1) show that all three variants had good imputation quality in the two cohorts (R ^2^>0.6 and info>0.7 for MAF<1% variants ^16^). However, we note that for rare variants these metrics may not be completely fit for purpose, as whilst the quality of imputation may look very good across all individuals, if the quality is poor for individuals with rare genotypes, we may have poor quality data on the most informative individuals. Therefore, we further investigated where exactly the discordance is observed between KASP genotyped and HRC imputed genotypes on an individual basis in the ALSPAC data. 

For each individual *FLG* genotype there is very little discordance in genotypes between the two methods (0.3% for R501X and <0.1% for R2447X and S3247X) amongst the 15,550 individuals with data from both, i.e. the vast majority fall in the concordant shaded cells of Table 2a–c. A potential limitation is that *FLG* null alleles are disproportionately represented in the individuals without confident calls in the imputed data (shown in missing rows of Table 2) as compared with the direct genotyping. Therefore, a proportion of likely true *FLG* mutation carriers would be excluded if using a ‘best-guess’ imputed data approach or more measurement error may be introduced if a dosage or probability imputed data approach is used.

**Table 2.** Concordance of *FLG* genotypes between KASP genotyping and HRC imputation in ALSPAC.

a. R501X	KASP genotypes			
Imputed genotypes	+/+	+/-	-/-	missing
+/+	14408	33	0	185
+/-	12	394	0	18
-/-	0	0	1	2
missing	240	237	3	17

b. R2447X	KASP genotypes			
Imputed genotypes	+/+	+/-	-/-	missing
+/+	15125	13	0	188
+/-	0	54	0	8
-/-	0	0	0	0
missing	76	69	0	17

c. S3247X	KASP genotypes			
Imputed genotypes	+/+	+/-	-/-	missing
+/+	15198	2	0	221
+/-	2	38	0	1
-/-	0	0	0	0
missing	38	50	0	0

d. Combined null genotype	3 KASP genotypes			4 KASP genotypes		
Imputed genotypes	No *FLG* mutations	1 or 2 *FLG* mutations	missing	No *FLG* mutations	1 or 2 *FLG* mutations	missing
No *FLG* mutations	13797	47	422	12879	699	687
1 or 2 *FLG* mutations	13	468	47	13	460	55
missing	359	351	46	342	358	56

For each variant, +/+ refers to the common wild type genotype (i.e. no mutations), +/- refers to heterozygotes (i.e individual with one mutation at this variant) and -/- refers to rare homozygote (i.e. both copies of the variant are mutated). The 3 individual mutations are also collapsed into a combined null genotype variable (part d), where individuals are stratified into those with no *FLG* null mutations and those with one or two *FLG* null mutations. In the KASP ^TM^ genotyped data this collapsing has been carried out for the 3 mutations that are available in the imputed data (“3 KASP genotypes”) and repeated also including the 2282del4 mutation (“4 KASP genotypes”) to show the impact of this variant being unavailable in the imputed data. Individuals are included as ‘missing’ if genotyping by both methods was attempted but failed in one or both for some reason. For the imputed data this includes individuals for whom the estimated dosages are not within the thresholds set for making hard genotype calls. *FLG*, filaggrin; HRC, Haplotype Reference Consortium; KASP, Competitive (Kompetative) allele-specific PCR.

For R501X, the overall discordance between the KASP ^TM^ and imputed genotypes is only 0.3%, with 12 (<0.1%) genotyped as wildtype (no R501X mutations) by KASP ^TM^ called as heterozygotes following imputation and 33 (5%) genotyped as heterozygotes called wild type in imputation. However, 237 (36%) of those genotyped as heterozygotes and 3 (75%) of those genotyped as rare homozygotes at this SNP had missing genotypes when only confident calls are counted in the imputed data.

For R2447X, overall discordance is <0.1%, with no individuals genotyped as wildtype imputed as having a R2447X mutation and 13 (10%) genotyped as heterozygotes imputed as wildtype. However, 69 (51%) of those genotyped as heterozygotes at this SNP had missing genotypes when only confident calls are counted in the imputed data. R501X and R2247X represent the same sequence alteration occurring at different locations within the highly repetitive sequence of *FLG* exon 3 and this may in contribute to genotype and imputation missing data.

For S3247X, overall discordance is <0.1%, with only 2 (<0.1%) genotyped as wildtype imputed as heterozygotes and 2 (2%) genotyped as heterozygotes imputed as wildtype. However, 50 (56%) of those genotyped as heterozygotes at this SNP had missing genotypes when only confident calls are counted in the imputed data.

Considering *FLG* genotypes are often dichotomised into groups with 1 or 2 *FLG* null mutations versus wild type genotype for statistical analysis, we demonstrate that overall discordance for such a variable is small (0.4%), only 13 (<0.1%) genotyped as wild type were imputed to harbour at least one *FLG* null mutation and 47 (5%) with at least one *FLG* mull mutation in the genotyped data were imputed as wild type. However, as also seen on the individual mutation basis, a large proportion (351, 41%) of individuals genotyped to have at least one *FLG* mutation, had missing data when only confident calls are counted in the imputed data. Furthermore, when we consider that the 2282del4 *FLG* mutation is not available in the imputed data, greater discordance (5%) is seen between KASP ^TM^ genotyped data of all 4 mutations and the imputed data for 3 SNPs.

We investigated how the discordance (and missingness in the imputed data) affected the observed association with AD in ALSPAC. Only R501X and the combined *FLG* null genotype showed strong associations with AD when using the KASP ^TM^ genotyped data (p=2x10 ^-9^ and p=2x10 ^-10^, respectively, Table 3). The odds ratios were perhaps slightly attenuated in the imputed data (odds ratio 2.08 versus 2.22 for R501X and OR=2.05 versus 2.08 for combined *FLG* null genotype, with overlapping confidence intervals), but both were still strongly associated using the imputed data (p=6x10 ^-10^ and p=4x10 ^-7^, respectively). R2447X and S3247X associations, whilst in the expected direction, did not show evidence for association in either the genotyped or the imputed ASLPAC data (all p>0.05). However, the much larger UK Biobank sample showed strong evidence for associations between AD and the three individual *FLG* variants and *FLG* combined null genotype (p-values ranging from 7x10 ^-10^ to 5x10 ^-75^), despite the data being imputed.

**Table 3.** Comparison of associations between *FLG* null mutations and atopic dermatitis in ALSPAC (using KASP ^TM^ genotyping and HRC imputation) and in UK Biobank (using HRC imputation).

	Association with atopic dermatitis phenotype OR (CI), P-value, (number of individuals in analysis)		
	ALSPAC – KASP	ALSPAC – imputed	UK Biobank - imputed
R501X	2.22 (1.72 to 2.88), p=2x10 ^-9^ (N=5,094)	2.08 (1.62 to 2.67), p=6x10 ^-10^ (N=5,155)	2.01 (1.86 to 2.16), p=5x10 ^-75^ (N=336,988)
R2447X	1.71 (1.00 to 2.92), p=0.051 (N=5,111)	1.42 (0.81 to 2.50), p=0.145 (N=5,155)	1.84 (1.58 to 2.15), p=9x10 ^-15^ (N=336,988)
S3247X	1.81 (0.89 to 3.68), 0.101 (N=5,110)	1.52 (0.74 to 3.10), p=0.193 (N=5,155)	1.75 (1.46 to 2.09), p=7x10 ^-10^ (N=336,988)
Combined null genotype (excluding 2282del4)	2.08 (1.66 to 2.61), p=2x10 ^-10^ (N=5,019)	2.05 (1.55 to 2.70), p=4x10 ^-7^ (N=4,924)	1.85 (1.72 to 1.99), p=7x10 ^-62^ (N=328,996)
Combined null genotype (including 2282del4)	1.96 (1.63 to 2.35), p=3x10 ^-13^ (N=4,934)	NA	NA

Results given are odds ratios (OR) and confidence intervals (CI) with the minor allele as the effect allele. The imputed analyses of individual genotypes use genotype probabilities or dosage to account for uncertainty in the genotype calls, the combined null genotype analyses use hard calls as defined in the methods. *FLG*, filaggrin; HRC, Haplotype Reference Consortium; KASP, Competitive (Kompetative) allele-specific PCR; NA, not applicable.

Our analyses have demonstrated that whilst some error is likely to be present in HRC imputed *FLG* variants, this method of calling *FLG* null genotypes in large population cohorts (where genome-wide imputation is readily available but bespoke genotyping is less often available and costly to obtain) is likely to be sufficient for many studies. Whilst there is likely to be some data missing-not-at-random (MNAR), when this is related only to exposure (so actual *FLG* status in this case) and confounders, but NOT the outcome (as seems likely in this case), then the exposure coefficient in a linear or logistic regression is unbiased ^17^. However, measurement error in a variable will lower power to detect associations and could bias the association towards the null. Therefore, whilst the coefficient estimate may not be reliable, the large sample sizes of cohorts such as ALSPAC and UK Biobank increase power sufficiently to allow detection of associations, as demonstrated by the very strong evidence seen for associations between AD and *FLG* variants in UK Biobank.

In our comparison, the UK Biobank suffers from an additional limitation that AD is likely to have been defined with more measurement error than it is in ALSPAC because in UK Biobank AD is a self-reported phenotype with recall bias or hospital statistic, whilst the participants in ALSPAC underwent longitudinal assessments (details in the Online Methods). However, despite the measurement error in both AD phenotype and *FLG* genotype, there is good evidence for the expected associations, in the expected direction (although probably with effect sizes that are biased somewhat towards the null).

Here, we have only assessed imputation using the HRC (r1.1 ^5^, or the combined HRC-UK10K reference used by UKBiobank ^11^) panel and so cannot comment directly on the utility of *FLG* imputations using other reference panels. But as HRC is the most advanced imputation panel developed to date, it is likely that previous imputation panels give less reliable genotype calls for these variants. The imputation quality of any SNP is also determined by the genotyping chip used in that study and particularly the density (and quality) of genotyping of SNPs in linkage disequilibrium with the variants of interest. ALSPAC was genotyped on the Illumina HumanHap550 quad array (children) or the Illumina human660W-quad array, and UK Biobank was genotyped on Applied Biosystems UK Biobank Axiom Array ^11^. Imputation from other genotyping chips (particularly those with markedly different properties) might need further validation. However, in general the imputation quality score of a variant of interest gives some information on how reliably that variant is imputed.

A limitation of our work is that it focussed on UK population data in which the prevalence of *FLG* null mutations has been extensively studied. The 3 mutations studied in our work vary in prevalence across different populations even within Europe. The lower prevalence of these mutations and a greater diversity in SNP genotypes in many African and Asian populations means the application of imputation requires further testing.

Whilst we have demonstrated that HRC imputation can provide adequate genotype calling for the most common *FLG* variants in large studies, we would still recommend caution if utilising such data. Imputation quality statistics should certainly be assessed, and we would also recommend the use of a positive-control analysis such as the associations with AD that we use in this study.

## Data availability

ALSPAC data access is through a system of managed open access. The steps below highlight how to apply for access to the data included in this research article and all other ALSPAC data. The datasets presented in this article are linked to ALSPAC project number B1533, please quote this project number during your application. The ALSPAC variable codes highlighted in the dataset descriptions can be used to specify required variables.

1. Please read the ALSPAC access policy which describes the process of accessing the data and samples in detail, and outlines the costs associated with doing so.

2. You may also find it useful to browse our fully searchable research proposals database, which lists all research projects that have been approved since April 2011.

3. Please submit your research proposal for consideration by the ALSPAC Executive Committee. You will receive a response within 10 working days to advise you whether your proposal has been approved.

If you have any questions about accessing data, please email alspac-data@bristol.ac.uk.

The study website also contains details of all the data that is available through a fully searchable data dictionary.

We used data from the UK Biobank resource under application number 10074 for this work. All *bona fide* researchers can apply to use the UK Biobank resource for health-related research that is in the public interest. Further information on the application process is available from the UK Biobank website.
