# Supplementary material for: Imputation provides an opportunity to study filaggrin ( FLG) null mutations in large population cohorts that lack bespoke genotyping
Source: Wellcome Open Res. 2024 May 20;7:36. Originally published 2022 Feb 1. [Version 2] doi: 10.12688/wellcomeopenres.17657.2 (PMC11301132; doi:10.12688/wellcomeopenres.17657.2)
Supplement: Peer review history

# Reviewer report: Reviewer response for version 2

**DOI:** 10.21956/wellcomeopenres.23964.r107080

**Published:** 2024-11-13

**Authors:** Luba M Pardo

**Affiliations:** 1 Erasmus University Medical Center, Rotterdam, The Netherlands

Review

 In this report Paternoster L et al present the results of a comparative allele frequencies analysis of four common *FLG* null mutations (R501X, R2447X and S3247X) profiled using genotyping KASP ^tm^ essays, with these derived from imputation analysis in two well-known large population-based studies (ALSPAC and UK Biobank). The authors also carried out logistic regression analysis to test the association of atopic dermatitis with the genotyped *FLG* null mutations and compared the effect sizes in ALSPAC and UK Biobank to. The imputed datasets were already available and were done using the HRC reference for the imputation. The authors found comparable allele frequencies between the genotyped and the imputed FLG-null mutations (only 3 mutations were available in the imputed datasets), with slightly lower frequencies in the imputed datasets. Imputation called rates for both population-datasets. The authors performed association analysis between the FLG-null mutations and AD per population-datasets and found increased risk of AD versus controls in these carrying at least 1 (null mutation). The effect size was stronger for the ALSPAC genotyped data versus this of the imputed ALSPAC dataset, but similar to this of the imputed UKBiobank dataset. This suggests that larger samples sizes can compensate for the uncertainty of imputation for rare variants as well. Authors concluded that imputed data appears could be used to analyse selected FLG null mutations.  

 Main comments.

 Paper is well-written, statistical analysis is clear as well as conclusions. The paper is relevant since it shows that in large enough datasets specific rare variants could have enough imputation quality for analysis. I only have one comment. The authors only used four known mutations but recent literature shows that other FLG-null mutations are likely to occur (PMID: 35042220). This may be relevant since in this report people without these 4 mutations were called as negative, which may decrease the strength of the associations.  I think the authors should also discuss this in the report.

Is the work clearly and accurately presented and does it cite the current literature?

Yes

If applicable, is the statistical analysis and its interpretation appropriate?

Yes

Are all the source data underlying the results available to ensure full reproducibility?

Yes

Is the study design appropriate and is the work technically sound?

Yes

Are the conclusions drawn adequately supported by the results?

Yes

Are sufficient details of methods and analysis provided to allow replication by others?

Yes

Reviewer Expertise:

genetic epidemiology. epidemiology of skin diseases.

I confirm that I have read this submission and believe that I have an appropriate level of expertise to confirm that it is of an acceptable scientific standard.

# Reviewer report: Reviewer response for version 2

**DOI:** 10.21956/wellcomeopenres.23964.r83819

**Published:** 2024-08-05

**Authors:** Cristina De Guzman Strong

**Affiliations:** 1 Department of Dermatology, Henry Ford Cancer Institute, Henry Ford Health, Michigan, USA

I have no further comments. Very nice paper.

Is the work clearly and accurately presented and does it cite the current literature?

Yes

If applicable, is the statistical analysis and its interpretation appropriate?

I cannot comment. A qualified statistician is required.

Are all the source data underlying the results available to ensure full reproducibility?

No source data required

Is the study design appropriate and is the work technically sound?

Yes

Are the conclusions drawn adequately supported by the results?

Yes

Are sufficient details of methods and analysis provided to allow replication by others?

Partly

Reviewer Expertise:

Human Genetics

I confirm that I have read this submission and believe that I have an appropriate level of expertise to confirm that it is of an acceptable scientific standard.

# Reviewer report: Reviewer response for version 1

**DOI:** 10.21956/wellcomeopenres.19533.r57780

**Published:** 2023-06-08

**Authors:** Cristina De Guzman Strong

**Affiliations:** 1 Department of Dermatology, Henry Ford Cancer Institute, Henry Ford Health, Michigan, USA

The manuscript by Paternoster et al provides a comprehensive study using ALSPAC and UK Biobank genotypes and imputation for three *FLG* null mutations to assert that HRC imputed data for UK-population-based genetic analysis can be used to identify specific *FLG* null mutations. As R501X, R2247X, and S3247X are difficult to accurately genotype, imputation circumvents this problem.  There are only minor concerns that need clarifications and a moderate concern for the calculations of discordance in Table 2.

1. In the Abstract’s 1 ^st^ sentence – please state the type of low frequency mutations? Null?
2. In the Abstract’s Methods – Nomenclature of the substitution variants needs to be consistent with varnomen.hgvs.org. See Sequence Variant Nomenclature (hgvs.org) for nonsense variants, ie R501*. This applies to the rest of the manuscript as well.
3. In the Abstract’s Conclusions, please list selected *FLG* null mutations.
4. Provide more details for KASP assays.
5. Please provide reference for ShapeIt v2.
6. Please provide reference for SNPTEST under “Associations between genotypes and AD in ALSPAC”.
7. It is not clear how the discordances of 0.3%, 0.1% and 0.1% for R501*, R2447* and S3247*, respectively, are calculated. I tried to calculate myself but perhaps I am missing a few details. Hence, I highlight that sufficient details of methods and analysis provided to allow replication by others are partly addressed and specific to this point. 

Is the work clearly and accurately presented and does it cite the current literature?

Yes

If applicable, is the statistical analysis and its interpretation appropriate?

I cannot comment. A qualified statistician is required.

Are all the source data underlying the results available to ensure full reproducibility?

No source data required

Is the study design appropriate and is the work technically sound?

Yes

Are the conclusions drawn adequately supported by the results?

Yes

Are sufficient details of methods and analysis provided to allow replication by others?

Partly

Reviewer Expertise:

Human Genetics

I confirm that I have read this submission and believe that I have an appropriate level of expertise to confirm that it is of an acceptable scientific standard, however I have significant reservations, as outlined above.

# Response

**Published:** 2024-04-20

**Authors:** Ashley Budu-Aggrey

**Affiliations:** University of Bristol, UK

**Reviewer Comment:**

 The manuscript by Paternoster et al provides a comprehensive study using ALSPAC and UK Biobank genotypes and imputation for three *FLG* null mutations to assert that HRC imputed data for UK-population-based genetic analysis can be used to identify specific *FLG* null mutations. As R501X, R2247X, and S3247X are difficult to accurately genotype, imputation circumvents this problem.  There are only minor concerns that need clarifications and a moderate concern for the calculations of discordance in Table 2.  

1. In the Abstract’s 1 ^st^ sentence – please state the type of low frequency mutations? Null?

***Author Response:*** **This sentence has been edited to refer to null *FLG* mutations**

**Reviewer Comment:**

1. In the Abstract’s Methods – Nomenclature of the substitution variants needs to be consistent with varnomen.hgvs.org. See Sequence Variant Nomenclature (hgvs.org) for nonsense variants, ie R501*. This applies to the rest of the manuscript as well.

***Author Response:*** **The nomenclature of the substitution variants have been updated**  

**Reviewer Comment:**

1. In the Abstract’s Conclusions, please list selected *FLG* null mutations.

***Author Response:*** **The *FLG* null mutations have now been listed.**    

**Reviewer Comment:**

1. Provide more details for KASP assays.

***Author Response:*** **Further details of the method have been included.**  

**Reviewer Comment:**

1. Please provide reference for ShapeIt v2.

***Author Response:*** **This reference has now been included**  

**Reviewer Comment:**

1. Please provide reference for SNPTEST under “Associations between genotypes and AD in ALSPAC”.

***Author Response:*** **This reference has now been included**

**Reviewer Comment:**

1. It is not clear how the discordances of 0.3%, 0.1% and 0.1% for R501*, R2447* and S3247*, respectively, are calculated. I tried to calculate myself but perhaps I am missing a few details. Hence, I highlight that sufficient details of methods and analysis provided to allow replication by others are partly addressed and specific to this point. 

***Author Response:*** **The discordances were calculated by taking the number of concordant genotypes away from the total number of genotypes assessed, dividing this by the total number of genotypes assessed and multiplying by 100. This has now been added to the main text.**

# Reviewer report: Reviewer response for version 1

**DOI:** 10.21956/wellcomeopenres.19533.r49578

**Published:** 2022-04-11

**Authors:** Hansjörg Baurecht

**Affiliations:** 1 Department of Epidemiology and Preventive Medicine, University of Regensburg, Regensburg, Germany

In this study, the ALSPAC and UK Biobank cohorts were used to investigate whether non-genotyped *FLG* null mutations can be imputed with high quality using the established imputation methodology used in genome-wide association studies (GWAS). The authors conclude that imputation based on the Haplotype Reference Consortium (HRC) resulted in an adequate determination of three of the most common *FLG* null mutations with discordance of 0.3% in UK populations.

- In general, the manuscript is well written. All technical details regarding genotyping, imputation and quality control of each study are clearly explained. The methods used are also very sound and state of the art.
-  A major problem of this study is that the *FLG* deletion 2282del4 cannot be detected by imputation. This deletion is very common among the four most common genotyped *FLG* mutations in European populations. Although this drawback is discussed in the Results and Discussion section, it should also be mentioned in the abstract.
- It would have been interesting to see if imputation based on other reference panels would give results of similar quality. This is raised as a limitation of the study and is not a major issue given that HRC is the most developed imputation reference panel.
- It would be helpful for the reader to see the proportion of concordance/discordance presented in Table 2.

**Minor comments:**

- Methods - ALSPAC cohort, page 4, 3rd paragraph: the imputation method used on the Michigan imputation server should be mentioned.
- Please harmonize the spelling of the name of the ShapeIT algorithm, which is "ShapeIT (v2.r644)" and "ShapeIt v2" on page 4, 3rd paragraph, and "SHAPEIT2" in the 9th paragraph.
- Please spell out UK10K on its first occurrence and add it to the abbreviations.
- Please spell out PCA on its first occurrence and add it to the abbreviations.

Is the work clearly and accurately presented and does it cite the current literature?

Yes

If applicable, is the statistical analysis and its interpretation appropriate?

Yes

Are all the source data underlying the results available to ensure full reproducibility?

Yes

Is the study design appropriate and is the work technically sound?

Yes

Are the conclusions drawn adequately supported by the results?

Yes

Are sufficient details of methods and analysis provided to allow replication by others?

Yes

Reviewer Expertise:

genetic epidemiology, epidemiology, atopic dermatitis research

I confirm that I have read this submission and believe that I have an appropriate level of expertise to confirm that it is of an acceptable scientific standard.

# Response

**Published:** 2024-04-20

**Authors:** Ashley Budu-Aggrey

**Affiliations:** University of Bristol, UK

**Reviewer Comment:**

 In this study, the ALSPAC and UK Biobank cohorts were used to investigate whether non-genotyped *FLG* null mutations can be imputed with high quality using the established imputation methodology used in genome-wide association studies (GWAS). The authors conclude that imputation based on the Haplotype Reference Consortium (HRC) resulted in an adequate determination of three of the most common *FLG* null mutations with discordance of 0.3% in UK populations.

- In general, the manuscript is well written. All technical details regarding genotyping, imputation and quality control of each study are clearly explained. The methods used are also very sound and state of the art.
-  A major problem of this study is that the *FLG* deletion 2282del4 cannot be detected by imputation. This deletion is very common among the four most common genotyped *FLG* mutations in European populations. Although this drawback is discussed in the Results and Discussion section, it should also be mentioned in the abstract.

***Author Response: *The abstract now mentions that we investigated the three *FLG* null mutations that could be detected by imputation**

**Reviewer Comment: **

- It would have been interesting to see if imputation based on other reference panels would give results of similar quality. This is raised as a limitation of the study and is not a major issue given that HRC is the most developed imputation reference panel.
- It would be helpful for the reader to see the proportion of concordance/discordance presented in Table 2.

***Author Response: *The discordances have now been included in Table 2**

**Reviewer Comment:**

**Minor comments:**

1. Methods - ALSPAC cohort, page 4, 3rd paragraph: the imputation method used on the Michigan imputation server should be mentioned.

***Author Response: *We have mentioned that imputation was performed using the MACH algorithm**

**Reviewer Comment:**

1. Please harmonize the spelling of the name of the ShapeIT algorithm, which is "ShapeIT (v2.r644)" and "ShapeIt v2" on page 4, 3rd paragraph, and "SHAPEIT2" in the 9th paragraph.

***Author Response: *This has now been harmonized to “ShapeIt v2”**

**Reviewer Comment:**

1. Please spell out UK10K on its first occurrence and add it to the abbreviations.

***Author Response: *This has been spelt out and added to the abbreviations list**

**Reviewer Comment:**

1. Please spell out PCA on its first occurrence and add it to the abbreviations.

***Author Response: *This has been spelt out and added to the abbreviations list**
